# The HIT Study—The Hydroxychloroquine Effect in the Treatment of Patients with Age-Related Macular Degeneration: A Randomized Controlled Trial

**DOI:** 10.3390/medicina59030551

**Published:** 2023-03-11

**Authors:** Tal Yahalomi, Yoav Pikkel, Roee Arnon, Dafi Porat, Joseph Pikkel

**Affiliations:** 1 Department of Ophthalmology, Faculty of Health Sciences, Samson Assuta Ashdod Hospital, Ben-Gurion University of the Negev, Beersheba 84105, Israel; 2 Plastic and Reconstructive Surgery, Rambam Health Care Campus, Haifa 3109601, Israel; 3 Rabin Medical Center, Petah Tikva 49100, Israel; 4 Kittner Skin Cancer Screening & Research Institute, Sheba Medical Center, Ramat Gan 5200100, Israel

**Keywords:** age-related macular degeneration, plaquenil, hydroxychloroquine, drusen, optical coherence tomography, visual acuity, central macular thickness

## Abstract

*Background and Objectives:* Decreased age-related macular degeneration (AMD) has been reported in individuals with rheumatoid arthritis treated with hydroxychloroquine (HCQ, plaquenil). *Materials and Methods:* In a randomized controlled trial with a parallel study design, we assessed visual acuity, central macular thickness measured with macular optical coherence tomography (OCT), and the number and size of drusen, following treatment with HCQ or a placebo in individuals with AMD. The patients received a daily dosage of 400 mg hydroxychloroquine (study group) or placebo (control group) during 12 months, and underwent complete ophthalmic examinations at 3, 6, 9, 12 and 24 months after initiation of treatment. Results: Of the 110 patients who were randomized to the treatment groups, 46 (29 females) in the study group and 50 (29 females) in the control group completed the study. The study group showed less visual acuity deterioration at two-year follow-up than did the control group (−0.03 ± 0.07 vs. −0.07 ± 0.07, *p* = 0.027). At two years after treatment initiation, the mean number of drusen per eye was lower for ARDS2 (8.1 vs. 12.3, *p* = 0.045) in the study group, compared to the control group. Compared to the control group, the proportion of eyes with increased drusen growth was smaller for both ARDS2 and ARDS3 drusen in the study group, and the proportion of the total drusen with growth was smaller for the study group as well: 32/46 eyes (70%) vs. 40/50 eyes (80%). Drusen volume growth, as calculated by the area and height measured with macular OCT, was also more reduced in the study than the control group (0.20 ± 0.15 vs. 0.23 ± 0.16 mm^4^, *p* = 0.05). None of the participants showed HCQ toxicity or adverse effects. Conclusion: Among patients with AMD, visual deterioration, the growth and the amount of drusen formation at two years after treatment initiation was less among those treated with HCQ than with a placebo. In this study, there was a negative association between HCQ treatment and wet AMD development.

## 1. Introduction

Age-related macular degeneration (AMD) is considered the third leading cause of blindness worldwide; the global prevalence is almost 20 million people [1]. Populations of European ancestry have shown particular increases in AMD incidence, especially after age 75 years [2]. Considering the aging population, the number of people with AMD worldwide has been estimated to rise to 288 million by 2040, with Asia bearing the heaviest burden [2].

**Figure 1:**
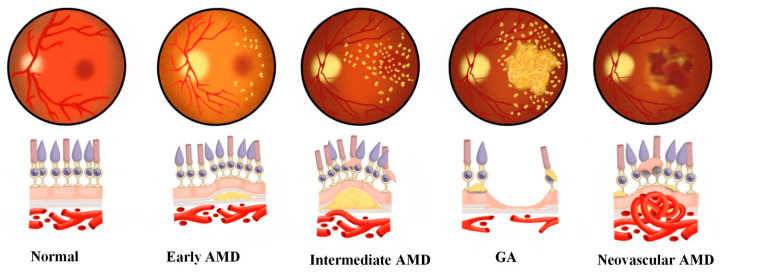

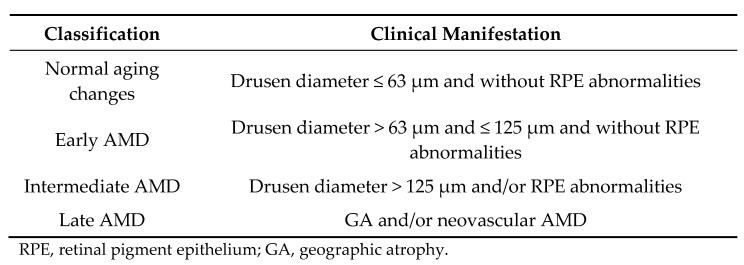
The Beckman clinical classification of age-related macular degeneration (AMD) [4].

AMD is characterized by the gradual degeneration of the retina's macular area. This results in central vision impairment, including metamorphopsia, decreased visual acuity and vision loss. Patients feel varying reductions in their quality of life, depending on the severity of their disease [3]. The disease is categorized into three clinical stages: early, intermediate and advanced AMD [4]. The appearance of drusen is the first clinical sign of early AMD (Figure 1). Medium and large drusen are defined as larger than 63 and 125 μm in diameter, respectively. The intermediate and advanced stages of AMD are responsible for central vision loss. Geographic atrophy (GA) and neovascular AMD are the two types of advanced AMD. GA is defined by the gradual degeneration of the retinal pigment epithelium (RPE), photoreceptor layer and choroidal capillaries in the macula region, which results in progressive vision loss over time [5]. The treatment of exudative wet AMD has been revolutionized by the combination of anti-vascular endothelial growth factor (VEGF) therapy and optical coherence tomography [5]. Anti-VEGF medications have improved the prognosis for wet AMD patients; however, even with treatment, some individuals still have debilitating visual impairments. Those treatments are also associated with a significant financial burden for individuals and the healthcare system because of their high cost and the requirement for regular follow-up therapies and consultations [5].

Hydroxychloroquine (HCQ) is commonly used to treat various indications such as autoimmune illnesses (e.g., rheumatoid arthritis), and also for malaria prevention. McGeer and Sibley first reported that their patients with rheumatoid arthritis treated with HCQ were relatively protected from the development of AMD [6], and that HCQ could confer a protective measure against AMD. McGeer and Sibley assumed that a possible explanation for the protective effect of HCQ could be the anti-inflammatory action of the drug, as the inflammatory component of macular degeneration is well known [6]. While some clinical and animal model evidence indicates that HCQ might cause systemic oxidative stress [7,8,9,10], this does not rule out the possibility that HCQ might also have a localized retinal antioxidative effect [11,12]. Moreover, HCQ was found to reduce migration and angiogenic activity of endothelial cells [12]. The mechanisms reported above could imply a protective role of HCQ in preventing AMD progression. A serious side effect in the use of HCQ is its retinal toxicity, which has long been known [13]. However, as we discuss below, only cumulative dosage is evident for retinal toxicity, and a meticulous follow-up with patients minimizes the risk of macular toxicity.

The Hydroxychloroquine effect In the Treatment of patients with age-related macular degeneration study (The HIT study) is a randomized controlled trial. We compared, in individuals diagnosed with AMD, the number of drusen, growth of drusen and visual acuity two years after initiation of one-year treatment with HCQ or a placebo.

## 2. Patients and Methods

### 2.1. Study Characteristics

This is a double-blind, randomized controlled trial of parallel design, with 1:1 allocation. Inclusion criteria were AMD with persistent drusen, categorized according to ARDS categories 2 and 3, without GA or prior ocular surgery, and consent to participate. ARDS category 2 was defined as <63 µmin diameter or non-extensive intermediate drusen of 63–125 µm diameter, and ARDS category 3 as extensive intermediate large drusen ≥125 µm diameter [14]. Patients were enrolled in the study while admitted to the ophthalmology department at Ziv Medical Center, northern Israel. Exclusion criteria were known diabetes mellitus, hypertensive retinopathy before the treatment or observed at the initial examination, GA or any prior ocular disease, and previous ocular surgical treatments including intravitreal injections or laser treatments. No changes were made to the trial protocol after commencement of the study.

Patients who met the initial inclusion criteria were enrolled in the study. The participants were randomly assigned to the study or control group by computer randomization. The physician who registered the participants to the groups using serial numbers did not take further part in the study.

### 2.2. Treatment Regime

The participants were instructed to take one untagged pill daily for 12 months. Containers of pills were allocated by patient serial numbers, with the pharmacy blind to the content of the pills. Pills for the study group contained 400 mg of HCQ (plaquenil). Placebo pills for the control group contained calcium. Patients who did not comply with the treatment given, did not attend the follow-up examination or withdrew their consent were excluded from the analysis.

### 2.3. Follow-Up Time Line

All the participants underwent a complete ophthalmic examination, including visual acuity (recorded as decimal score), intraocular pressure, anterior and posterior segment inspection and optical coherence tomography (OCT) for measuring central macular thickness. Since one of the first signs of macular toxicity of HCQ is a reduction of red light sensitivity, during the follow-ups, all participants underwent visual field testing conducted as macular visual fields of red/white. Macular fields were evaluated using the macular perimeter software in the Macular OCT HS-100 (Canon Inc, Tokyo, Japan). The number and volume of existing drusen were assessed by red-free fundus imaging. Participants were examined at 3, 6, 9, 12 and 24 months following the initiation of treatment. The follow-up was extended to 12 months following completion of treatment (up to two years after the beginning of the study), to ascertain the lack of HCQ-induced retinal maculopathy or another influence or adverse effect. All participants underwent extensive rheumatologic assessment and follow-ups, and any systemic adverse effects were also assessed.

### 2.4. Evaluation of Drusen and Macular Appearance

**Figure 2:**
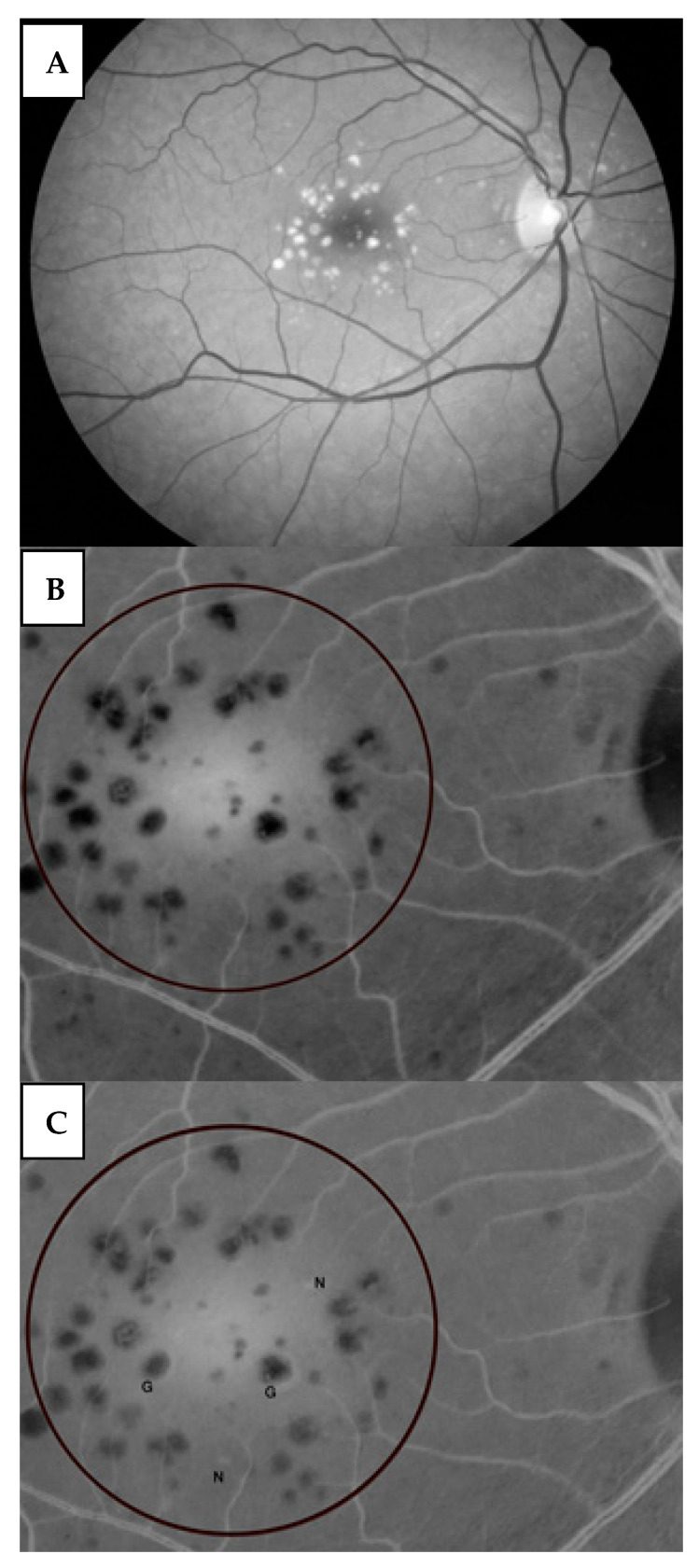
Red-free fundus snap-shot. (**A**) All the participants underwent an initial red-free fundus snap-shot. The number and volume of existing drusen were further assessed by subsequent red-free fundus imaging (Top). (**B**) A circle of an area of 1.5 optic discs around the macula was drawn, and the whole picture was transformed into a negative image (Middle). (**C**) Super-position of the final snap-shot (positive) on the initial snap-shot (negative). Drusen with a white halo are drusen of increased size (marked G). New drusen are represented by a white spot (marked N).

We used red-free images to evaluate the growth of new drusen or increased size of known drusen: Each participant underwent an initial red-free fundus snap-shot (Figure 2A). A marked circle of a 1.5 optic disc diameter around the macula was drawn, and drusen within this marked area were counted (Figure 2B,C). At one-year follow-up, another red-free snap-shot was taken, and the drusen were counted similarly. To detect drusen that were not visible in the red-free technique and to assess drusen volume in addition to their number and area growth, drusen were assessed using the Macular OCT HS-100 (Canon Inc, Tokyo, Japan). Drusen were also assessed with OCT en face and with an OCT B photograph of 12-line radial scanning centered on the foveal zone 10 × 10 mm in size (Canon Inc, Tokyo, Japan). Drusen volume was calculated by measuring area size and height. At follow-up examinations, drusen number and volume were not assessed. However, at the final examination, we used the “follow-up mode” to scan the macular zone in the same place, and the number and volume of drusen were measured as in the baseline examination. Drusen number and volume were assessed separately by two examiners. If a difference between their assessments exceeded 5%, a third independent examiner re-evaluated the photographs and made the final decision. Drusen volume was measured according to the technique that was described by Schlanitz et al. Drusen volume growth was calculated as the area and height measured with macular OCT; 7.5% was considered to be growth in volume [15,16]. Drusen volumes with red-free or macular OCT photographs were all calculated in a masked fashion, using a standardized protocol (two examiners and a third examiner when needed). As the natural course for drusen is initial progression in size and subsequent regression, we measured drusen regression in addition to their growth, in both the study and control groups, referring to drusen type as defined by the ARDS study group [4] (Figure 1).

### 2.5. Clinical and Demographic Characteristics

Demographic characteristics, visual acuity, retinal width, and initial and final follow-up drusen data were collected and analyzed.

### 2.6. Statistical Analysis

For continuous variables, arithmetic means and standard deviations were calculated. For categorical variables, relative frequencies were calculated. The Mann–Whitney non-parametric test was applied to examine differences between the study groups for quantitative parameters; all the hypotheses were one-tail. Pearson chi-square and Fisher’s exact test were applied to examine correlations between the study and control groups of the categorical parameters. The sample size was determined by statistician consultation. A *p*-value of 5% or less was considered statistically significant. The data were analyzed using the SPSS version 20.0.2 (SPSS Inc., Chicago, IL, USA). The study was approved by the local bioethical review committee.

### 2.7. Sample Size

The sample size was calculated using a significance level of 5% and power of 80%, with an acceptable mean difference of 0.05 logMAR (half-line) [17] and standard deviation of 0.07. The actual difference between the means was assumed to be 0.1. The estimated sample size was 25 for each group. The calculation was performed using PASS 2022 Power Analysis and Sample Size Software (2022) (NCSS, LLC. Kaysville, UT, USA, ncss.com/software/pass; accessed on 1st of January 2023). We decided to recruit twice the number required by the sample size calculation, due to an estimated loss to follow-up of 50%.

## 3. Results

### 3.1. Clinical and Demographic Characteristics

**Figure 3:**
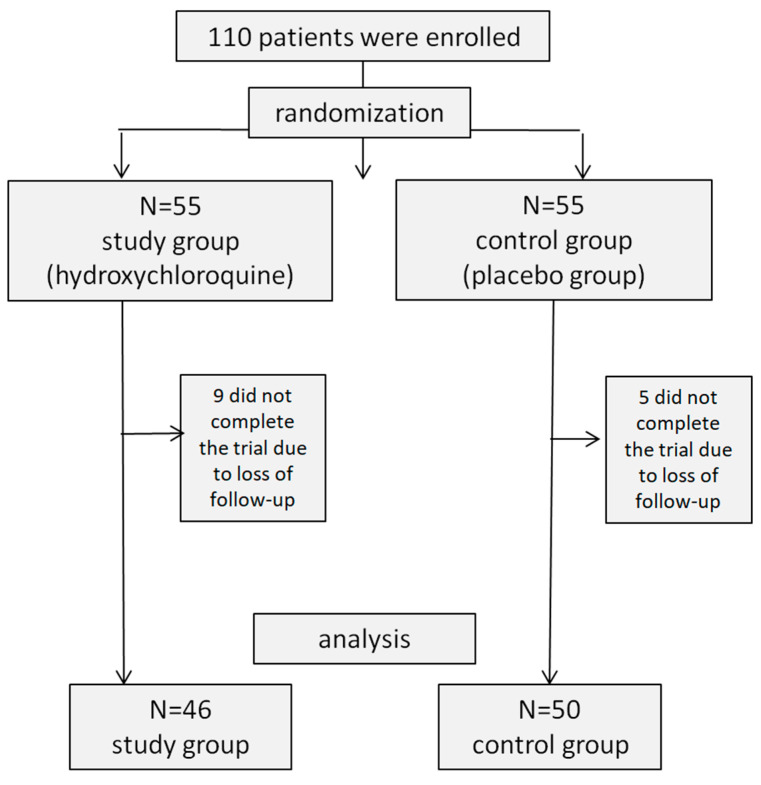
Flow Diagram of the study’s participants. Of the 110 patients who enrolled in the study, 55 were randomized to the study group, and 55 to the control group. Fourteen patients did not complete the trial due to loss of follow-up. Forty-six patients in the study group and fifty in the control group completed the study.

Of the 110 patients who enrolled in the study, 55 were randomized to the study group and 55 to the control group. Fourteen patients did not complete the trial due to loss of follow-up. Forty-six patients in the study group and fifty in the control group completed the study (Figure 3). The proportions of females in the two groups were similar: 29/46 (63%) and 29/50 (58%), respectively, *p* = 0.79. The mean ages were also similar: 76.0 ± 5.4 and 77.5 ± 4.5 years, respectively, *p* = 0.38. The two groups did not differ in their ocular characteristics, including the number of drusen in each eye (*p* = 0.28 in the right eye, *p* = 0.45 in the left eye, *p* = 0.79 in both eyes), primary visual acuity (*p* = 0.89), primary central macular thickness observed with macular OCT (*p* = 0.79), or the number of patients complaining of metamorphosis (*p* = 1.3) (Table 1).

### 3.2. Visual Acuity

Visual acuity deterioration at two-year follow-up compared to baseline was less in the study than in the control group (−0.03 ± 0.07 vs. −0.07 ± 0.07, *p* = 0.027).

### 3.3. Central Macular Thickness

The difference between the study and control groups, in increased central macular thickness at two-year follow-up compared to baseline, was not statistically significant: 8.9 ± 21.6 and 13.2 ± 9.5, respectively, *p* = 0.21.

### 3.4. Drusen Number and Size

One year following the end of the treatment, the mean number of drusen per eye was lower in the study than the control group for ARDS type 2 (ARDS2) (8.1 vs. 12.3, *p* = 0.045), and similar between the groups for ARDS type 3 (ARDS3) (7.4 vs. 9.2, *p* = 0.87). At two-year follow-up, for both the study and control groups, the proportion of eyes (divided by the number of patients) with increased drusen size was lower for both ARDS2 and ARDS3. For the respective groups, drusen size was increased for ARDS2 in 24/46 (52%) vs. 29/50 (58%) eyes, *p* = 0.034; and for ARDS3 in 11/46 (24%) vs. 16/50 (32%) eyes, *p* = 0.045. The proportion of eyes with total drusen growth was also lower in the study than in the control group: 32/46 (70%) vs. 40/50 (80%) eyes, *p* = 0.051.

The study and control groups showed similar proportions of eyes with regression of drusen size, as measured with macular OCT at the final examination, one year following the end of treatment. For ARDS2, these proportions for the respective groups were 3/46 (7%) vs. 4/50 (8%) eyes, *p* = 0.773. For ARDS3, the proportions were 11/46 (24%) vs. 9/50 (18%) eyes, *p* = 0.43. In the final examination, drusen volume growth was lower in the study than in the control group: 0.20 (±0.15) vs. 0.23 (±0.16) mm^4^, *p* = 0.058 (Table 2).

### 3.5. Safety and Adverse Effects

None of the participants showed HCQ toxicity manifested by cornea verticillata or changes in the red-to-white macular field of vision. Perimetric follow-up was performed by using the MP1 OCT and showed that none of the participants developed absolute or relative scotoma, and MP1 general-reduction-of-light threshold was not detected in any of the participants of the study group nor in the control group during the two-year follow-up.

None of the participants in the study group developed GA or active choroidal neovascularization. None of the participants in the control group developed active AMD; however, one participant developed GA in both eyes. The trial ended, as planned, two years after commencement. None of the trial participants had any systemic adverse effects.

## 4. Discussion

The HIT study is the first study to address the effect of HCQ on the clinical and imaging characteristics of AMD. The main finding of this double-blind randomized controlled trial was less visual acuity deterioration at one-year follow-up among participants treated with HCQ than those treated with a placebo. Moreover, compared to the control group, the mean number of drusen per eye was lower for ARDS2 in the study group. For the study group, compared to the control group, the proportion of eyes with increased drusen growth was less, for both ARDS2 and ARDS3 drusen, and the total drusen growth was less. Drusen volume growth, as calculated by the area and height measured with macular OCT, was also more reduced in the study than the control group. None of the participants showed HCQ toxicity or adverse effects.

Currently, no treatment can reverse AMD, due to the nature of retinal nerve insult. Thus, a preventive treatment could change the entire natural course of this disease. The HIT study examined the possibility that HCQ could be such an agent.

Previous studies have examined means of preventing the development of AMD. Some studies focused on the correlation of retinal thinning or thickness with the development and progression of non-exudative (dry) AMD [18,19,20,21]. Others focused on ocular manifestations in early AMD that are associated with the choroid [22]. Investigations of structural and functional characteristics of AMD have revealed correlations between retinal sensitivity and drusen, and between the sensitivity and integrity of the outer segment junction [2]. Previous studies investigated the influence of drusen development in reducing the progression of AMD. Klein et al. reported that patients treated with statins were less likely to have soft drusen or late AMD during a five-year follow-up period [23]. Vavvas et al. reported regression of drusen and improved vision among patients treated with statins [24]. Drusen accumulation has been linked to advancing AMD and has been considered a clinical therapeutic target in early AMD [14]. Drusen size and volume were found to correlate with the development of GA and choroidal neovascularization [15].

Thus, if HCQ affects the development of drusen, it could stop the deterioration of the disease without long-term damage. The rationale of the current study emerged from the work of McGeer and Sibley, who showed that patients with rheumatoid arthritis who were treated with HCQ were relatively protected from the development of AMD [8], and from the autopsy study by Berenstein and Ginsberg [9]. McGeer and Sibley reported an AMD incidence of 0.2% (3/993 patients) among people with rheumatoid arthritis, far lower than the incidences reported in the Beaver Dam eye study (3.4%, 67 of 1955 people) [10], the Rotterdam study (2.5%, 101/4071 people) and the Blue Mountains Survey (3.2%, 63/1950 people) [16]. In the age group of 75 years and older, McGeer and Sibley found that the prevalence of AMD was 0.4% (2/497) among individuals with rheumatoid arthritis who were treated with HCQ. This compares with a prevalence of 3.7% (516/13,900) in the UK survey [17], among a general population of the same age. McGeer and Sibley hypothesized that their results could be explained by the long-term anti-inflammatory and antioxidative effect on the retina in the use of HCQ. As drusen are long known to be the predisposing sign for the development of AMD [18], in this study, we examined the effect of HCQ on drusen formation and volume.

The retina is generally considered one of the most oxygen-consuming tissues in the human body, consuming even more oxygen per weight than the brain [25]. While the retina requires substantial energy, it produces reactive oxygen species (ROS) such as superoxide, hydroxyl radical, hydrogen peroxide and singlet oxygen as typical metabolic byproducts. Yet, ROS production that exceeds the capacity of antioxidant systems causes oxidative stress, which renders the retina vulnerable to oxidative damage [26]. With age, antioxidants degrade, increasing intracellular ROS, which leads to cellular and mitochondrial malfunction. Adenosine triphosphate (ATP) generation depends on mitochondria, where ROS are primarily formed [27]. Overproduction of ROS causes pathologic states and cellular malfunction in numerous tissues [28]. For instance, oxidative DNA damage induces transcriptome alterations related to aging by causing telomere shortening, DNA methylation, histone de-acetylation and mitochondrial dysfunction [29]. By inhibiting the 26S proteasome, ROS also cause buildup of ubiquitinated proteins. Oxidized proteins eventually aggregate inside cells as a result of insufficient protein degradation [30]. Late-stage AMD has been characterized by RPE cell loss and photoreceptor impairment due to oxidative stress [31]. According to in vitro research, short-wavelength light promotes ROS generation in the mitochondria [32]. The features of the unique sources of retinal ROS formation and high oxygen consumption imply that oxidative damage has an important role in the development of AMD.

Soft or large drusen may form, consequent to the formation of localized detachments of basal linear or laminar deposits; both these deposits reflect granular material collecting between the RPE and its basement membrane or outside the RPE's basement membrane [33]. The size and density of drusen are important determinants of disease development [34,35]. Drusen and disciform lesions have been detected together with AMD for over one century [36]. Drusen are considered a hallmark of AMD, and large, soft or confluent drusen are more likely to indicate AMD rather than normal aging [37]. In 2013, the Beckman Initiative for Macular Research Categorization Committee recommended a new clinical classification method for AMD to address this issue [4] (Figure 1). The Beckman AMD categorization system classifies AMD into three stages: early, intermediate and late (GA or neovascular AMD). The appearance of medium-sized drusen (diameter > 63 and 125 μm) without apparent impairment of visual function characterizes early-stage AMD. The presence of large drusen (diameter > 125 μm) and/or anomalies in the RPE is characterized as intermediate-stage AMD. Late-stage AMD (advanced AMD) is classified into two clinical subtypes: GA (non-exudative or dry AMD) and neovascular AMD (wet AMD). GA is characterized by the permanent loss of RPE and photoreceptor cells, which results in reduced visual function. Thus, drusen have a significant role in AMD development. 

The demographic and pre-study clinical characteristics were similar for the study and control groups, reflecting good randomization. Comparing the final examination results (at two years from the beginning of the study, which was 12 months after treatment cessation), the mean number of new drusen formations was less in the study group, and fewer drusen grew in size. At the two-year follow-up, there was no difference in metamorphopsia between the groups. In both groups, the central macular was somewhat thicker at two-year follow-up, more so in the control group, though the difference between the groups was not statistically significant. At the final follow-up, the most significant difference between the groups was in visual acuity. Though visual acuity decreased in both groups, in the control group, this reduction was significantly greater than in the study group, yet only by 0.04 logMar.

As drusen are considered to predispose to AMD, the main objective of this study was to show that HCQ treatment may reduce drusen production. Our study suggests that HCQ may have a protective effect against the development of inactive AMD (dry AMD), and also active AMD (wet AMD). We could not distinguish whether this effect is due to increased thickening of the retina outer band (RPE and Bruch’s membrane), as we suggested in our previous study [7], or due to another reason, such as an anti-inflammatory effect, as has been suggested by others [8,9].

**Figure 4:**
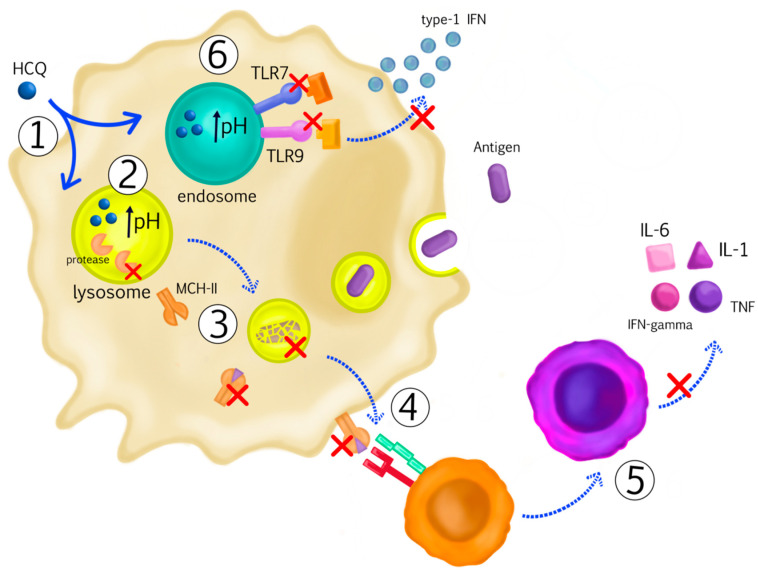
Suggested mechanisms of the anti-inflammatory and immunomodulatory effects of HCQ. 1. HCQ has a hydrophilic nature, can pass through cell membranes easily and accumulates in intracellular vesicles, including lysosomes, endosomes and autophagosomes. 2. Raising the pH in the acidic vesicles precludes proper functioning of vesicular enzymes. 3. HCQ prevents the presentation of peptides for major histocompatibility complex (MHC)-II, by interfering with the conversion of antigens to peptides in antigen-presenting cells. Hence, extracellular material may accumulate less under the retina. 4. HCQ prevents peptides from interacting with MHC-II in the loading compartment of acidic endosomes on MHC-II. 5. HCQ inhibits cytokine release by interfering with MHC-II-related autoantigen presentation to cluster of differentiation (CD) 4+ T-cells, via antigen-presenting cells. This prevents CD4+ T-cells from activating B-cells. HCQ causes autoreactive T-cells from undergoing apoptosis and obstructs B-cells from processing antigens. The result is impaired function and production of cytokines, including interleukin [IL]-1, IL-6, interferon-gamma, tumor necrosis factor and B-cell activating factor. 6. The binding of immune complexes to TLR7 and TLR9 results in the downstream stimulation of type-1 interferon transcription. Type-1 interferons trigger the generation of additional cytokines by activating T-cells, B-cells, natural killer cells, myeloid dendritic cells and monocytes. The binding of TLR7 and TLR9 to the immune complexes is directly inhibited by the accumulation of HCQ in endosomes that contain TLR7 and TLR9. HCQ can potentially hinder the processing of TLRs by raising the pH of the endosome. Thus, HCQ reduces the transcription of type-1 interferons by interfering with TLR7 and TLR9 signaling; this has immunomodulatory and anti-inflammatory effects.

The precise mechanism underlying the anti-inflammatory and immunomodulatory effects of HCQ remains unknown, even though the drug is effective in treating several autoimmune and inflammatory illnesses, including systemic lupus erythematosus and rheumatoid arthritis. Due to its hydrophilic nature, HCQ can pass through cell membranes easily and accumulates in intracellular vesicles, including lysosomes, endosomes and autophagosomes. Raising the pH in these acidic vesicles precludes proper functioning of vesicular enzymes (such as proteases) [38]. HCQ prevents the presentation of peptides for major histocompatibility complex (MHC)-II, by interfering with the conversion of antigens to peptides in antigen-presenting cells [39,40]. Hence, extracellular material may accumulate less under the retina. This might explain the phenomena we observed in this study, namely lesser growth and lesser new formation of drusen in the participants who were treated with HCQ. Furthermore, HCQ may prevent peptides from interacting with MHC-II in the loading compartment of acidic endosomes on MHC-II. HCQ also inhibits cytokine release by interfering with MHC-II-related autoantigen presentation to cluster of differentiation (CD) 4+ T-cells, via antigen-presenting cells. This prevents CD4+ T-cells from activating B-cells. HCQ causes autoreactive T-cells from undergoing apoptosis and obstructs B-cells from processing antigens. The result is impaired function and production of cytokines, including interleukin [IL]-1, IL-6, interferon-gamma, tumor necrosis factor and B-cell activating factor [40,41]. Inhibiting toll-like receptor (TLR) signaling pathways is one of the immunomodulatory mechanisms of HCQ that has received attention [42]. Immune complexes with DNA or RNA bind to the Fc-gamma receptor-II of the plasmacytoid dendritic cell and internalize to endosomes. The latter have intracellular TLR7 and TLR9 that identify single-stranded RNA and DNA, respectively. Through the myeloid differentiation primary response protein 88, the binding of immune complexes to TLR7 and TLR9 results in the downstream stimulation of type-1 interferon transcription. Type-1 interferons trigger the generation of additional cytokines by activating T-cells, B-cells, natural killer cells, myeloid dendritic cells and monocytes [39,43]. The binding of TLR7 and TLR9 to the immune complexes is directly inhibited by the accumulation of HCQ in endosomes that contain TLR7 and TLR9. HCQ can potentially hinder the processing of TLRs by raising the pH of the endosome [44]. Thus, HCQ reduces the transcription of type-1 interferons by interfering with TLR7 and TLR9 signaling; this has immunomodulatory and anti-inflammatory effects [39,43] (Figure 4).

A main concern when treating patients with HCQ is retinal toxicity [24,25]. This is the most devastating side effect of HCQ, and when severe, can even cause blindness. None of the participants of this study developed any sign of HCQ toxicity. Furthermore, none developed corneal sediments (cornea verticillata), changes in macular red/white visual field (examined in each participant once in three months) or toxic maculopathy. Among the several factors that have been associated with this risk, dosage seems the most important. The data are inconclusive as to whether daily intake or cumulative dosage is most significant. Recent studies indicate that cumulative dosage may have a greater effect than daily dosage [26]. The current recommendations are not to exceed a cumulative dosage of 1000 g. Several online calculators available for doctors and patients provide the safe duration for using the drug, following input of body weight and daily use of HCQ. Accordingly, a person weighing 80 kg can safely take the drug in the dosage we used (400 mg per day) for almost eight years. Nonetheless, individuals using the drug should be followed for toxic retinopathy. We strongly recommend a follow-up routine for patients using HCQ, in addition to a thorough examination and medical history anamnesis before starting treatment [27].

Further studies are needed to explain the impact of HCQ on the retina and its protective effect against AMD development, whether due to thickening of the retinal outer band, an anti-inflammatory effect or something else. This study was composed of a single institute and comprised a relatively small number of participants. Treatment with HCQ and follow-up periods were relatively short. Adverse effects of a continuous therapy may first present only several years after initiation of treatment.

## 5. Conclusions

This study showed less growth and less drusen formation in individuals with AMD treated with HCQ than in a control group, at two-year follow-up. In this study, there was a negative association between HCQ treatment and wet AMD development. Our findings suggest that prophylactic treatment with HCQ might hinder the progression of AMD and effectively eliminate this disease as a cause of blindness among the elderly. Further investigation is needed to establish our conclusions and to identify the optimal pharmacological regime for individuals with AMD. Though previous studies suggested an anti-inflammatory effect of HCQ in preventing AMD, this prospective study showed reduced growth of drusen in patients treated with HCQ compared to a placebo. The latter process is recognized as a predisposing factor of AMD.

## Figures and Tables

**Table 1:** Demographic characteristics and baseline ocular characteristics of the study (hydroxychloroquine) and control (placebo) groups.

	Study (*n* = 46)		Control (*n* = 50)		
	*n*	Sd	*n*	Sd	*p*
Gender: Female (*n*,%)	29 (61.5%)		29 (59%)		0.79
Age (years)	77.5	4.5	76.0	5.4	0.38
Drusen number (mean) at presentation					
Right	14.1	1.5	13.8	1.4	0.28
Left	14.4	1.4	14.5	1.5	0.45
Both eyes	14.3	1.4	14.2	1.5	0.79
Primary visual acuity					
	0.2	0.01	0.18	0.011	0.89
Primary OCT central macular thickness					
	237	46	241	51	0.79

OCT: optical coherence tomography; Sd: standard deviation.

**Table 2:** Drusen volume growth as calculated by area and height, measured using optical coherence tomography.

	Study Group			Control Group			
	Mean Volume	SD	Range	Mean Volume	SD	Range	*p* Value
**Initial Examination**	0.18 mm^3^	0.17	0.009–0.6	0.19	0.16	0.008–0.7	0.89
**Final Examination**	0.20 mm^3^	0.15	0.01–0.65	0.23	0.16	0.011–0.73	0.038

## Data Availability

This study was registered in the United States National Library of Medicine Clinical Trials Registry CLINICALTRAILS.GOV–Identifier number: NCT01541449.
